# Supplementary material for: Cardiac digital twins at scale from MRI: Open tools and representative models from ~ 55000 UK Biobank participants
Source: PLoS One. 2025 Jul 15;20(7):e0327158. doi: 10.1371/journal.pone.0327158 (PMC12262899; doi:10.1371/journal.pone.0327158)
Supplement: S1 Table — (PDF) [file pone.0327158.s001.pdf]

Table 1. The dataset utilized from the UKBB.

UKBB field id	Description
31-0.0	Sex of participant
34-0.0	Year of birth of participant
52-0.0	Calendar month of birth of participant
53-2.0	Date of the first imaging visit
20208-2.0	Long axis heart images acquired at the first imaging visit
20209-2.0	Short axis heart images acquired at the first imaging visit
21001-2.0	Body mass index of participant at the first imaging visit
24100-2.0	LV end diastolic volume at the first imaging visit. See [1, 2] for methodology.
24101-2.0	LV end systolic volume at the first imaging visit. See [1, 2] for methodology.
24103-2.0	LV ejection fraction at the first imaging visit. See [1, 2] for methodology.
24105-2.0	LV myocardial mass at the first imaging visit. See [1, 2] for methodology.
24106-2.0	RV end diastolic volume at the first imaging visit. See [1, 2] for methodology.
24107-2.0	RV end systolic volume at the first imaging visit. See [1, 2] for methodology.
24109-2.0	RV ejection fraction at the first imaging visit. See [1, 2] for methodology.
24110-2.0	LA maximum volume at the first imaging visit. See [1, 2] for methodology.
24111-2.0	LA minimum volume at the first imaging visit. See [1, 2] for methodology.
24114-2.0	RA maximum volume at the first imaging visit. See [1, 2] for methodology.
24115-2.0	RA minimum volume at the first imaging visit. See [1, 2] for methodology.
31065-2.0	RA maximum volume at the first imaging visit. See [3] for methodology.
31066-2.0	RA minimum volume at the first imaging visit. See [3] for methodology.
31075-2.0	LA maximum volume at the first imaging visit. See [3] for methodology.
31076-2.0	LA minimum volume at the first imaging visit. See [3] for methodology.
Return 2541	This return from study [3] contains various data derived from heart MRI, we used the manual segmentation files after converting from cvi42 format to segmentation masks.

References

1. Bai W, Sinclair M, Tarroni G, Oktay O, Rajchl M, Vaillant G, et al. Automated cardiovascular magnetic resonance image analysis with fully convolutional networks. Journal of Cardiovascular Magnetic Resonance. 2018;20(1):65. doi:https://doi.org/10.1186/s12968-018-0471-x.

2. Bai W, Suzuki H, Huang J, Francis C, Wang S, Tarroni G, et al. A population-based phenome-wide association study of cardiac and aortic structure and function. Nature medicine. 2020;26(10):1654–1662.

3. Petersen SE, Aung N, Sanghvi MM, Zemrak F, Fung K, Paiva JM, et al. Reference ranges for cardiac structure and function using cardiovascular magnetic resonance (CMR) in Caucasians from the UK Biobank population cohort. Journal of Cardiovascular Magnetic Resonance. 2016;19(1):18. doi:https://doi.org/10.1186/s12968-017-0327-9.
