# Supplementary material for: Cardiac digital twins at scale from MRI: Open tools and representative models from ~ 55000 UK Biobank participants
Source: PLoS One. 2025 Jul 15;20(7):e0327158. doi: 10.1371/journal.pone.0327158 (PMC12262899; doi:10.1371/journal.pone.0327158)
Supplement: S2 Table — A number of participants or meshes were removed at certain steps of the pipeline due to reasons such as missing views, failing automatic quality-control checks, failing to produce an output etc. This table details the reasons for removal and the number of participants or meshes remaining after these removals. (PDF) [file pone.0327158.s002.pdf]

Table 1. Number of subjects or meshes utilized at different steps of the pipeline.

Description	#
Initial number of subjects that have both LAX and SAX DICOMs	55835
Number of subjects that have all four views after DICOM to NIFTI conversion	54926
Number of subjects that pass contour quality-control	51549
Number of subjects for which ED surface meshes are successfully built	51358
Number of subjects with viable ED surface meshes after removal of outlier meshes	48993
Number of surface meshes used to create the average meshes after binning	46917
Number of initial average surface meshes	1428
Number of average meshes for which fibers and UVCs were successfully created	1423

A number of subjects or meshes were removed at certain steps of the pipeline due to reasons such as missing views, failing automatic quality-control checks, failing to produce an output etc. This table details the reasons for removal and the number of subjects or meshes remaining after these removals.
