# Supplementary figures and images for: Cardiac digital twins at scale from MRI: Open tools and representative models from ~ 55000 UK Biobank participants
Source: PLoS One. 2025 Jul 15;20(7):e0327158. doi: 10.1371/journal.pone.0327158 (PMC12262899; doi:10.1371/journal.pone.0327158)

nnUNet vs manual (n=731)

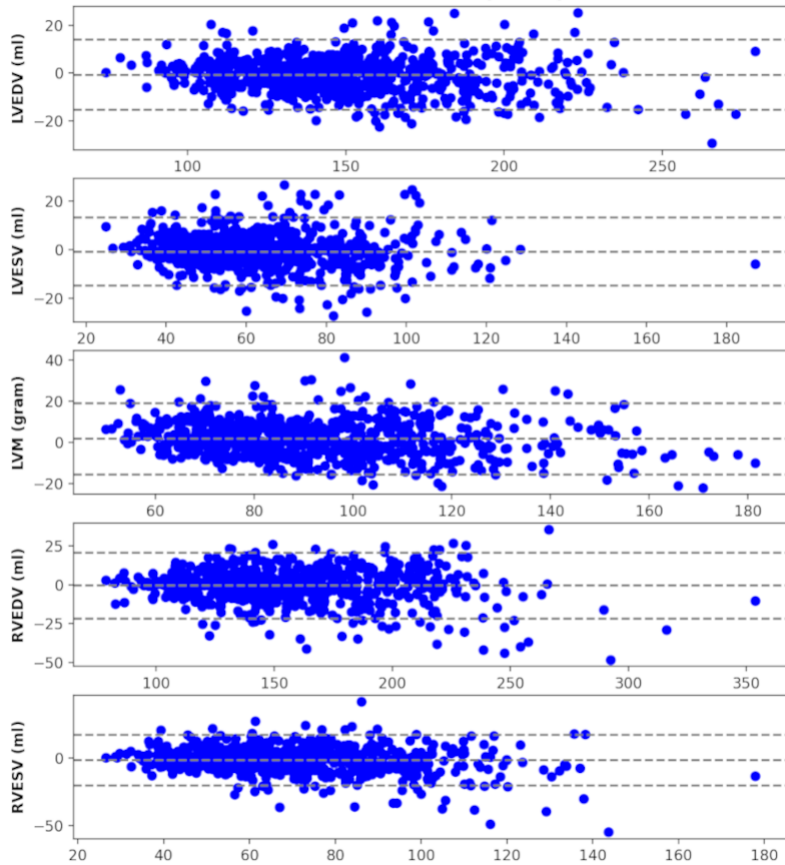

Mesh (Ours) vs manual (n=731)

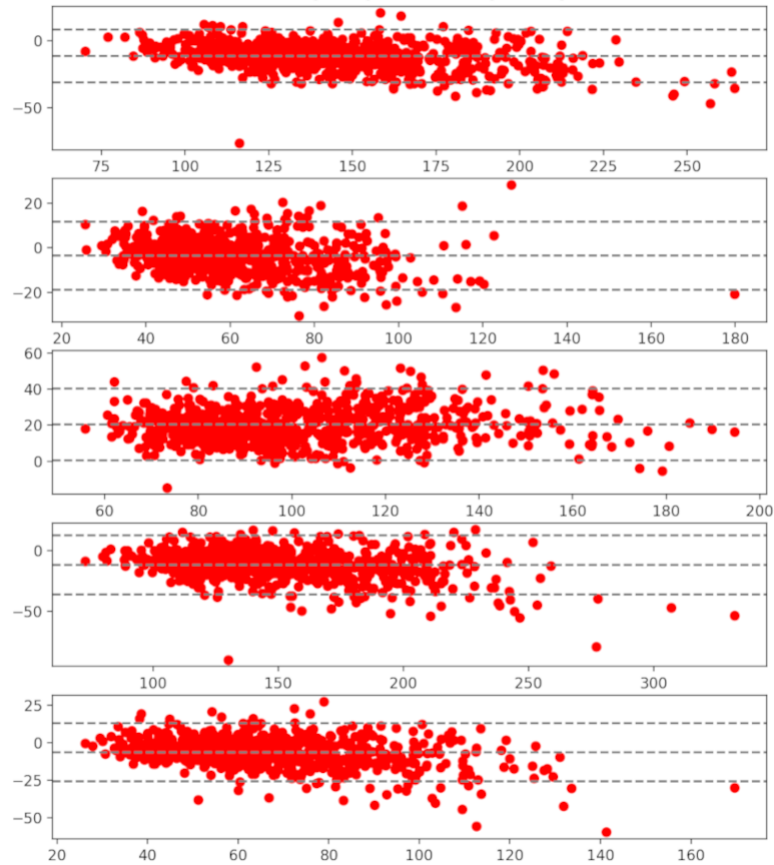

Supplement: S1 Fig — (PDF) [file pone.0327158.s003.pdf]
